# Alteration of hypnotic experience following transcranial electrical stimulation of the left prefrontal cortex

**DOI:** 10.1016/j.ijchp.2022.100346

**Published:** 2022-11-10

**Authors:** Rinaldo Livio Perri, Gloria Di Filippo

**Affiliations:** a University Niccolò Cusano, Rome, Italy; b De Sanctis Clinical Center (CCDS), Rome, Italy

**Keywords:** Hypnosis, Neurostimulation, Hypnotizability, Dorsolateral prefrontal cortex, Consciousness

## Abstract

**Background:**

Recent studies of neurostimulation reported alteration of hypnotizability and hypnotic phenomena after inhibition of the dorsolateral prefrontal cortex (DLPFC), but the different assessments of hypnosis and the stimulation parameters still left open many questions about the role of this brain region in hypnotizability. We aimed to administer inhibitory transcranial direct current stimulation (tDCS) over the left DLPFC to observe effects of stimulation on the hypnotic experience and the feeling of agency.

**Methods:**

a procedure of hypnotic induction with suggestions was repeated twice: before and after the unilateral cathodal tDCS over the left DLPFC. The experience was assessed through a phenomenological assessment of hypnosis and sense of agency in thirty-three participants randomly assigned to the sham or the active group.

**Results:**

active (inhibitory) tDCS enhanced the hypnotizability by 15.4% and altered a few dimensions of consciousness such as self-awareness and absorption. No changes emerged on the feeling of agency and pass rates for suggestions.

**Conclusions:**

tDCS reflects a promising tool to alter the hypnotic phenomena and the responsiveness to hypnotic procedures. Neurocognitive implications are discussed for the construct of hypnotizability as well as for the role of the left DLPFC in the dimensions of consciousness such as self-awareness.

## Introduction

Hypnosis is as a subjective experience of focused attention and reduced peripheral awareness which relies on several factors such as neurobiology, motivation, expectancy, relation, attention (Dienes et al., 2012; Facco, 2021; Pekala et al., 2017; Testoni et al., 2020). Connected to hypnosis is the construct of hypnotizability, which refers to the ability to experience suggested alterations in physiology, sensations, emotions, thoughts or behavior during hypnosis (Elkins et al., 2015). The experimental hypnosis is based on the assumption that the higher the hypnotizability the more intense the response to the hypnotic suggestions, and hypnotizability was also associated to clinical outcomes of the hypnotherapeutic interventions (e.g., Thompson et al. 2019). However, these should not be considered as strict relationships as they were not always confirmed (e.g. Perri et al. 2019), and the definition of hypnotizability such as its quantification are still under discussion in the literature (Dixon & Laurence, 1992; Perri, 2022). In fact, since its birth hypnosis has been linked to the concept of suggestibility, and the main scales used to assess hypnotizability in terms of behavioral responses to the hypnotist's suggestions (see Facco 2021 for a review), while emerging evidence describe hypnosis as a multidimensional phenomenon including both behavioral and experiential components (Acunzo & Terhune, 2021).

With the aim to improve the hypnotic responsiveness a few studies attempted to enhance the hypnotizability through psychological (see Lynn 2004 for a review) and pharmacological approaches (Bryant et al., 2012; Whalley & Brooks, 2009). For example, it was shown that that subjects initially scored as low hypnotizables became highs after receiving the Carleton Skills Training Program (CSTP; Bertrand et al. 1993) and that administration of oxytocin increased the hypnotic responsiveness (Bryant et al., 2012). More recently, the non-invasive brain stimulation (NIBS) techniques have been tested in this field as well. In particular, two studies (Coltheart et al., 2018; Dienes & Hutton, 2013) documented transient increases of responsiveness to hypnotic suggestions when disrupting the activity of the right or left dorsolateral prefrontal cortex (DLPFC) through the repetitive transcranial magnetic stimulation (rTMS). The same cortical areas have been stimulated by our group as well (Perri et al., 2022), which however adopted the transcranial direct current stimulation (tDCS). Moreover, unlike previous studies we measured the hypnotic experience through the Phenomenological Consciousness Inventory: the Hypnotic Assessment Procedure (PCI-HAP; Pekala et al., 2010) in order to assess the variations in consciousness in addition to the canonical measures of hypnotizability. Findings showed that the cathodal (inhibitory) tDCS of the left DLPFC (lDLPFC) reduced the volitional control by 30% and enhanced the hypnotic depth by 11% on the Hypnoidal State Score (HSS) of the PCI-HAP (see methods for details). The study confirmed the key-role of the DLPFC in the hypnotic phenomena and suggested the possibility to get relevant enhancements of hypnotizability through neurostimulation. However, the use of a bilateral tDCS montage did not allow to exclude the contribution of the right DLPFC (i.e., the return electrode site) on the observed results. In fact, as it was supposed that return electrode could have opposite physiological effects when put over the cranium (for review see DaSilva et al. 2011), it was possible that the concomitant activation of the rDLPFC contributed to the results or, at the opposite, that it limited the magnitude of the effects. In order to test these alternative hypotheses and aim for stronger results, in the present study we adopted an extracephalic tDCS montage to provide unilateral stimulation of the lDLPFC. Moreover, being the agency disruption one of the core aspect of the hypnotic phenomena (Polito et al., 2013), we also decided to adopt a specific measure of agency for each of the suggestions provided. Our hypothesis was that the reduced excitability of the lDLPFC could enhance the hypnotizability and allow for a more intense response on different kinds of hypnotic suggestions. In fact, changes in the DLPFC activity during hypnosis were associated with altered executive control (for a review see Landry et al. 2017), and deactivation of the left hemisphere seems to best reflect the reduction of peripheral awareness during neutral hypnosis (Dienes & Hutton, 2013; Perri et al., 2020b). The contribution of the DLPFC in the hypnotic phenomena was also emphasized by the COLD control theory (Dienes et al., 2012; Dienes & Perner, 2007), according to which hypnosis would consist in the inaccurate ‘higher order thoughts’ (HOTs) leading to the subjects being unaware of his/her intentions in motor and cognitive actions. As a consequence, hypnosis responses would be the result of the reduced awareness of the voluntary control (COLD control) and the left DLPFC was mainly associated to this metacognitive function (Dienes & Hutton, 2013).

## Materials and methods

### Participants

An *a priori* power analysis for the repeated measure, within-between interaction ANOVA design was done with the G*Power software (Faul et al., 2007) to determine the minimum sample size to detect an effect size (η^2^p) >0.14 (Cohen, 2013). The input effect size (*f* = 0.42) was determined using the automated direct method available in G*Power. The other parameters were the α error probability = 0.05, the power (1-β error probability) = 0.95, the number of groups = 2, the number of measurements = 2, the correlation among repeated measures = 0.50 and the ε = 1. Results of the power analysis indicated a minimum of 22 subjects with an actual power of 0.96.

Thirty-three healthy volunteers participated in this study. They were recruited from the student population at the Niccolò Cusano University. Subjects were randomly assigned to the sham (*N* = 18, 6 males, mean age = 23.5 ± 3.5) or the active group (*N* = 15, 6 males, mean age = 23.1 ± 6.4), and all of them were in their first hypnotic experience as revealed by the pre-assessment questionnaire of the PCI-HAP (see below for details). Before participating in the study, all participants gave their written informed consent. The procedures were approved by the ethical committee of the IRCCS Santa Lucia Foundation (Prot. CE/2024_029) and were in accordance with the ethical standards of the 1964 Declaration of Helsinki.

### Phenomenological hypnotic assessment: the PCI-HAP

Administration of the PCI-HAP includes the Pre-Assessment, the hypnotic procedure, the Post-Assessment and the PCI. The PCI is a 53-item self-reported questionnaire retrospectively assessing the phenomenological experience in reference to a specific stimulus condition during hypnosis (Pekala et al., 2010; Pekala & Kumar, 2007). Except for the imagery trial, the original protocol of the PCI-HAP includes only one hypnotic suggestion, that is the challenge suggestion of eye catalepsy. However, we have added two more items belonging to the category of motor (arm levitation) and cognitive-perceptual suggestions (taste hallucination). Overall, it is estimated that about 80% of people pass motor suggestions, about 50% challenge suggestions and 10% cognitive suggestions (Kallio & Ihamuotila, 1999; Perry et al., 1992). The intensity of the response to the hypnotic suggestions was self-rated during the post-assessment stage on a 0 (nothing) to 10 (extremely) scale (i.e., “how intense was the bitter sensation in the mouth?” and “how intense was the feeling of lightness in your arm?”).

The PCI explores the phenomenological experience through 14 minor and 12 major dimensions of consciousness, while the PCI-HAP scoring provides different domains such as the self-reported hypnotic depth (srHD) and the *Hypnoidal State Score* (HSS). The HSS is a measure of hypnotic depth that correlates about .60 (Forbes & Pekala, 1993; Pekala & Kumar, 1984) with scores on the Harvard Group Scale of hypnotic susceptibility (Shor & Orne, 1962). The HHS generates an estimate of Weitzenhoffer's conceptualization of “trance,” and it is based on a regression equation consisting for 10 of the PCI (sub)dimensions (see Table 1): the HSS may be the only quantifiable, phenomenological or “noetic” measure of “trance” available to date (Pekala, 2015; Pekala et al., 2017).

**Table 1:** Hypnoidal State Regression Equation used to generate the hypnoidal state score, HSS. Percentages indicate relative magnitude of the coefficient. Each PCI (sub)dimension is rated on a 0-6 scale, with 0 indicating "none or little," and 6 indicating "much or complete” (From Pekala & Kumar, 1984).

**PCI (Sub)Dimension**	**Coefficient**	**Relative Percentage**
Altered Experience	+.35	17%
Altered State	+.31	15%
Volitional Control	-.28	13%
Self-Awareness	-.27	13%
Rationality	+.23	11%
Absorption	+.19	9%
Memory	-.14	7%
Altered Time Sense	+.13	6%
Internal Dialogue	-.11	5%
Altered Body Image	-.07	3%

### Sense of agency rating scale (SOARS)

The SOARS is a 10-item scale that indexes subjective disruptions of agency in hypnosis (Polito et al., 2013). For each item, participants are asked to rate their level of agreement on a 7-point Likert scale from “strongly disagree” to “strongly agree”. SOARS has two factors: the first, Involuntariness, includes items such as “I felt that my experiences and actions were not caused by me” and reflects the experienced reduction in control over one's own actions that is characterised by an external locus of control. The second factor, Effortlessness, includes items such as “My experiences and actions occurred effortlessly” and reflects the perception of automaticity of actions. Participants answered three SOARS questionnaires: one for each of the suggestions administered during hypnosis: eye catalepsy, hand levitation, taste hallucination.

### tDCS and study design

**Fig. 1:**
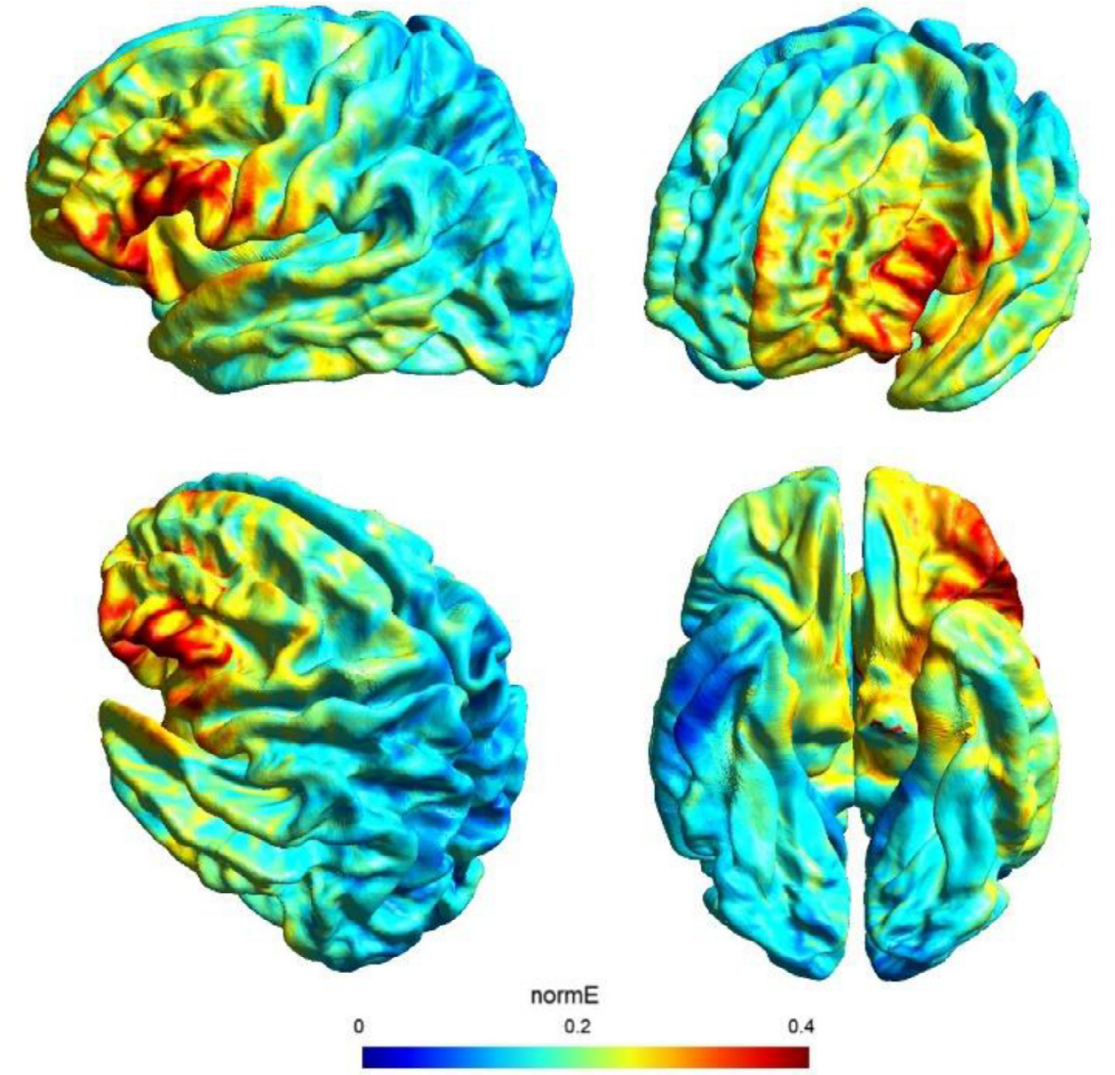
Electric field modeling for the F3/right shoulder tDCS montage. Simulation was performed with SimNIBS 3.2.

Direct current was transferred by a saline-soaked pair of surface sponge electrodes (25 cm^2^) and delivered by a battery-driven constant current stimulator in a randomized, sham-controlled protocol. We decided to adopt the same stimulation parameters of a previous study (see Perri et al., 2022 for more details on this choice) with a different montage in order to check for any differences of the bilateral vs unilateral stimulation of the DLPFC. In particular, we adopted a unilateral extracephalic tDCS montage with the target electrode over the left DLFPC (F3 site of the 10/20 system) and the return electrode over the right deltoid. The target electrode provided a negative current (cathodal stimulation) delivered by the software-based BrainStim stimulator (EMS srl, Bologna, Italy). For the active stimulation, the current intensity was gradually increased for 10 s at the beginning of the stimulation session (ramp up), delivered at -2.0 mA for 18 min and decreased for 10 s at the end of the session (ramp down) to diminish its perception. In the sham stimulation, the ramp up was delivered for 10 s until reaching -2.0 mA, the current was transferred for 7 s and was followed by a ramp down lasting 10 s. Then, after 18 min of no-stimulation, the ramp up-ramp down cycle was repeated at the end of the session. Potential adverse effects of tDCS were assessed by the experimenter at the end of each session using an interview inspired by Brunoni et al. (2011) questionnaire: none of the participants reported any significant adverse effect. Also, subjects were asked to guess the assigned group (active or sham) and identification was at chance level: this is not a perfect method of assessing sham blinding (Turner et al., 2021), but it should be noted that subjects were tDCS naïve and received only one stimulation, further reducing the possibility of being aware of the stimulation parameters. The simulation of the electric field cortical distribution was performed using SimNIBS 3.2 software (Thielscher et al., 2015): the present stimulation targeted the lateral portion of the left PFC probably corresponding to the middle frontal gyrus (MFG), as depicted in Fig. 1.

For participants of both groups, the experiment started with the administration of the PCI-HAP and the SOARS (pre-stimulation condition); then, the tDCS electrodes were mounted and the stimulation was provided; when finished, the PCI-HAP and SOARS procedure was repeated (post-stimulation condition). The whole experiment lasted about 110 min.

### Statistical analysis

Normal distribution of data was verified using the Shapiro-Wilk test, and all the scales of the PCI-HAP, the SOARS factors and the self-reports of the hypnotic suggestions were submitted to 2 × 2 RM-ANOVAs with Group (sham, active) and Session (pre-, post-stimulation) as independent and dependent factors, respectively. Results were corrected for multiple comparisons using the Fisher's Least Significant Difference (LSD) test, and the effect size was calculated as partial eta squared (η^2^p; ≥0.01, small effect; ≥0.06, moderate effect; ≥0.14, large effect; Cohen, 2013). Correlational analyses (Pearson's *r*) were performed between the SOARS and the perceived intensity for each suggestion, such as between the SOARS and the HSS (measure of hypnotizability). The overall α level was fixed at 0.05.

## Results

**Fig. 2:**
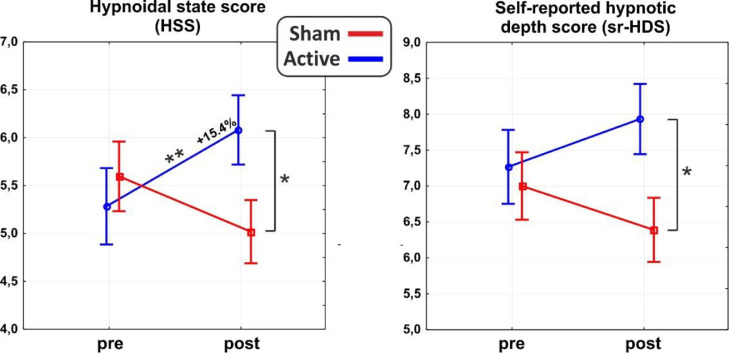
ANOVA on the Hypnoidal State Score (HSS; left) and self-rated Hypnotic Depth Score (sr-HDS; right). The active group increased both scores after the tDCS. *p < 0.05, **p < 0.01.

As for the main domains of the PCI-HAP, ANOVAs revealed significant interaction effects for the HSS (F_1,31_ = 10.8, *p* < 0.01, η^2^p = 0.26) and the self-reported hypnotic depth score (sr-HDS; F_1,31_ = 4.7, *p* < 0.5, η^2^p = 0.13). In particular, post hoc analysis on the HSS showed a significant difference between post-stimulation values of the active and sham group (*p* < 0.05), such as between the pre- (5.2±1.8) and the post-stimulation (6.0±1.4; *p* = 0.01) of the active group, reflecting an average increase of 15.4% in hypnotic depth. As for the sr-HDS, post hoc revealed a significant difference between post-stimulation values of active and sham group (*p* < 0.05). See Fig. 2 for a depiction of the results.

**Fig. 3:**
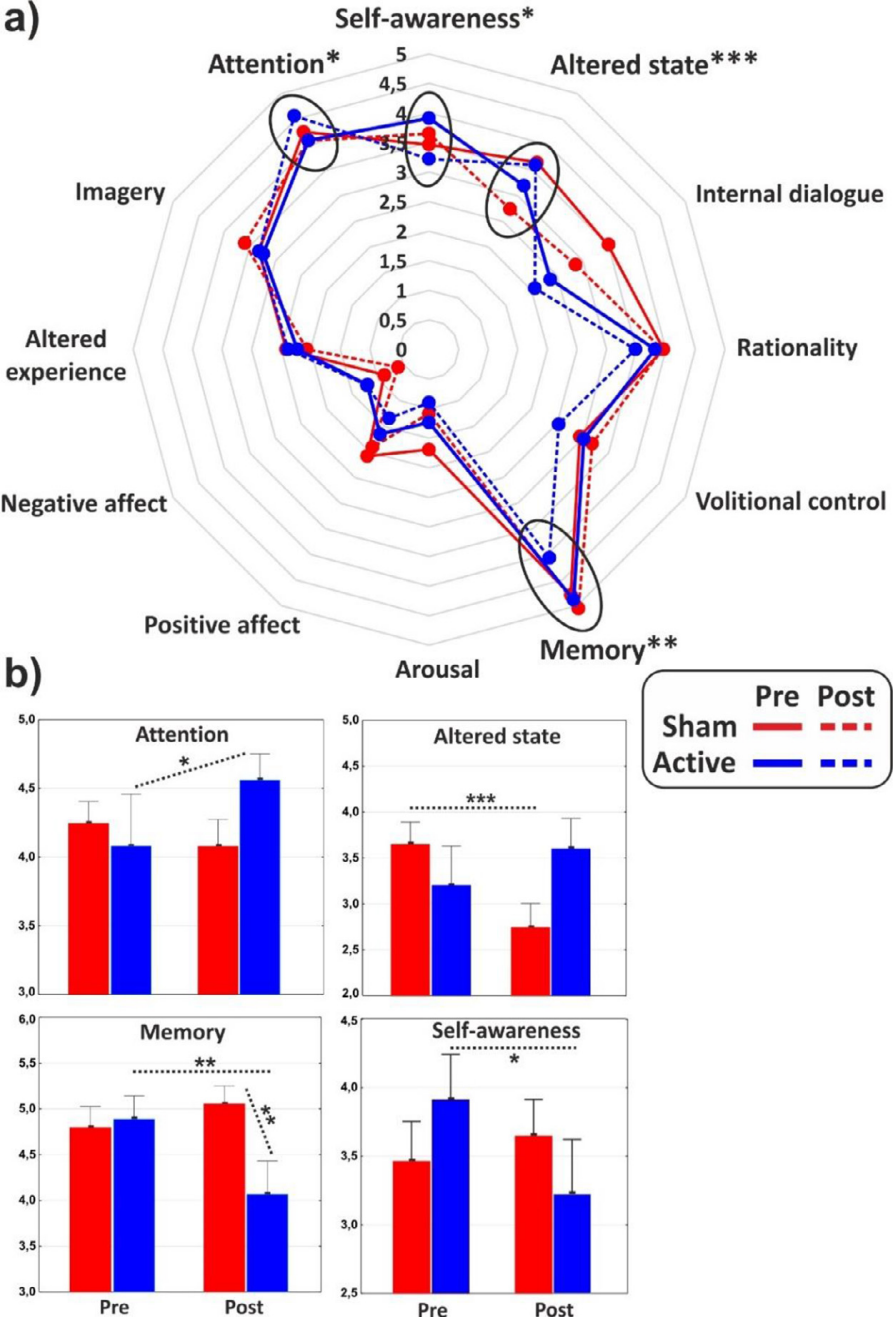
(a) Radar of the major dimensions of consciousness as identified by the PCI. (b) Histograms of the subdimensions significantly affected by the experimental conditions. Values of pre- and post-stimulation are reported for the active and the sham group. *<0.05, **p < 0.01, ***p < 0.001.

ANOVAs on the major dimensions of the PCI showed a significant interaction effect for the (sub)dimensions of Attention (F_1,31_ = 4.3, *p* < 0.05, η^2^p = 0.12), Memory (F_1,31_ = 7, p = 0.01, η^2^p = 0.18) and Altered state (F_1,31_ = 17.2, *p* < 0.001, η^2^p = 0.36), while the Self-awareness almost reached statistical significance (F_1,31_ = 3.4, *p* = 0.06). In particular, post hoc comparisons revealed that cathodal tDCS increased Attention (*p* < 0.05), while it reduced Memory (*p* < 0.01) and Self-Awareness (*p* < 0.05). Altered state decreased (*p* < 0.001) in the post-stimulation of the control group. All the PCI major dimensions are reported in Fig. 3 for the two groups in both conditions.

**Fig. 4:**
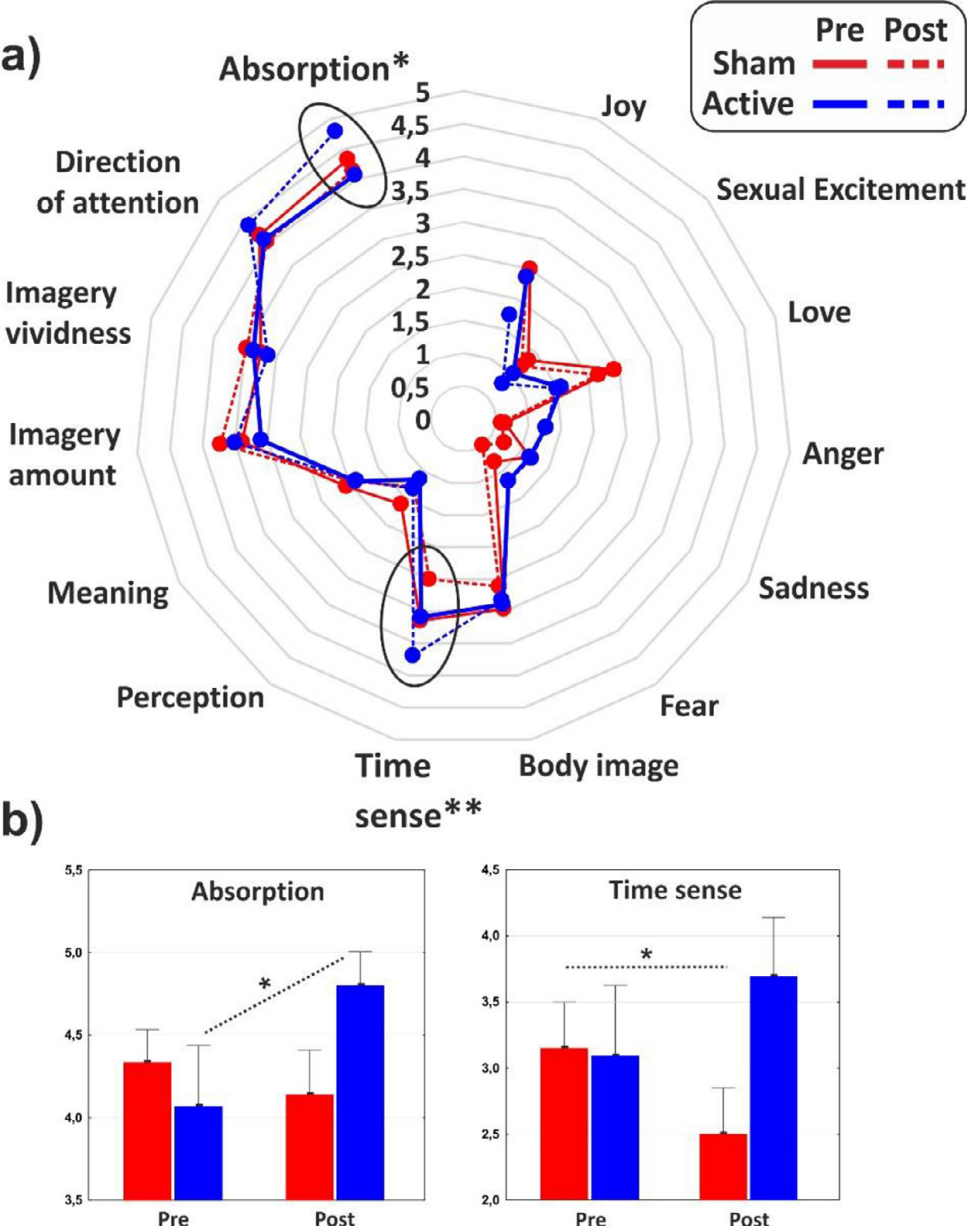
(a) Radar of the minor dimensions of consciousness as identified by the PCI. (b) Histograms of the subdimensions significantly affected by the experimental conditions. Values of pre- and post-stimulation are reported for the active and the sham group. *<0.05, **p < 0.01.

As for the minor dimensions of the PCI, significant effect of interaction emerged on the Absorption (F_1,31_ = 4.1, *p* < 0.05, η^2^p = 0.11) that increased as effect of the cathodal tDCS, and Time sense (F_1,31_ = 8, *p* < 0.01, η^2^p = 0.2) that decreased in the post-stimulation of the control group. The minor dimensions of the PCI are reported in Fig. 4 for the two groups in both conditions.

ANOVAs on the vividness of visual imagery, on the perceived intensity of the suggestions such on the SOARS tests did not reveal any effect of stimulation (all ps > 0.05). As for the correlational analyses, before the tDCS (pre-stimulation) significant results emerged between the HSS score and the involuntariness factor of the SOARS for all the suggestions rated: eye catalepsy (*r* = .56, *p* < 0.01), arm levitation (*r* = .55, *p* < 0.01) and taste hallucination (*r* = .44, *p* < 0.05). Also, the involuntariness of the SOARS correlated with the self-rated intensity of the eye catalepsy (*r* = .40, *p* = 0.05), while the effortless factor of the SOARS correlated with arm levitation (*r* = .42, *p* = 0.05) and taste hallucination (*r* = .41, *p* = 0.05). In the post-stimulation condition, all the self-rated suggestion-SOARS correlations remained significant, while the HSS correlations remained significant only with the SOARS of the eye catalepsy (*r* = .51, *p* < 0.01) and with its self-rating (*r* = .51, *p* < 0.01). See Table 2 for a summary of the correlational analysis.

**Table 2:** Correlations between the main variables considered in the study (Pearson's *r* are reported) for the pre- and the post-stimulation conditions. HSS: hypnoidal state score; SOARS inv and SOARS eff refer to the involuntariness and the effortlessness factor of the agency test, respectively; catalepsy, levitation and hallucination are referred to the eye catalepsy, arm levitation and taste hallucination suggestions, respectively. *p < 0.05, **p < 0.01, ***p < 0.001.

			Catalepsy		Levitation		Hallucination	
		HSS	SOARS inv	SOARS eff	SOARS inv	SOARS eff	SOARS inv	SOARS eff
Pre-stimulation	HSS	-	.56**	.08	.55**	.20	.44*	.03
	Self-rated catalepsy	.58**	.40*	.24	-	-	-	-
	Self-rated levitation	.17	-	-	-.03	.42*	-	-
	Self-rated hallucination	.00	-	-	-	-	.32	.41*
Post-stimulation	HSS	-	.51**	-.14	.21	-.17	.28	.24
	Self-rated catalepsy	.51**	.71***	-.38	-	-	-	-
	Self-rated levitation	-.21	-	-	.00	.81***	-	-
	Self-rated hallucination	.38	-	-	-	-	.36	.49**

## Discussion

Findings from the present study showed that the cathodal tDCS of the left DLPFC modified a few hypnotic phenomena and enhanced both the hypnotizability index (HSS) and the self-reported hypnotic depth (sr-HDS) of subjects selected regardless of their hypnotic abilities. At the opposite, and contrary to our hypothesis, the stimulation did not affect neither the responsiveness to the ideomotor, challenge and perceptual suggestions nor the associated feeling of agency.

The use of a retrospective instrument of phenomenological assessment (PCI-HAP) allowed us to identify the dimensions of consciousness significantly affected by the neurostimulation: they were the Attention (+11.8%), Memory (-16.8%), Self-awareness (-17.6%) and Absorption (+18.2%). Except from Attention, the other dimensions participate in the regression equation used to generate the hypnoidal state score (HSS; Pekala & Kumar, 1984) which was increased by 15.4% in the active group. The intensity and the effect size of the increase of hypnotizability further strengthened the results of a previous study (Perri et al., 2022) where a bilateral montage was adopted to stimulate the lDLPFC. However, unlike the previous investigation, the volition and the sense of agency were not affected by tDCS as revealed by the assessments of the hypnotic experience (PCI-HAP) and the response to hypnotic suggestions (SOARS). In our opinion, the reason of the unaltered sense of agency may lie in the electric field distribution of the tDCS montage. In fact, while the bilateral stimulation of the lDLPFC spread the current effects to the dorsal-medial cortical areas (for details see Perri et al., 2022) with unpredictable consequence on the right hemisphere (DaSilva et al., 2011), the extracephalic montage of the present study targeted a more focused and lateral region probably corresponding to the left middle frontal gyrus (MFG; see Fig. 1).

It is important to point out that the frontal lobe is a key-region of agency in the human brain together with parietal and subcortical regions (for a review see Haggard, 2017), and that the different portions of the frontal cortex subtend different aspects of agency. In particular, activity of the supplementary motor area (SMA) was associated with feeling of control and ownership of movements (Walsh et al., 2015), and it is recruited to predict the sensory consequences of an action such as to process agency error during its execution (Zito et al., 2020); the dorsal fronto-median cortex has been associated with explicit assignments of agency (Spengler et al., 2009), and the deactivation of the medial PFC described as a sign of the reduced engagement of the default network (DN), a brain network associated with internal attention and mind wandering reflecting the reduction of automatic thoughts in hypnosis (for a review see Landry et al. 2017); as for the DLPFC, its activation correlated with the self-rated level of hypnotic “depth” (Deeley et al., 2012), and it was associated with metacognition (Dienes & Perner, 2007), conscious judgments about the self (Miele et al., 2011) and action-selection processes (Haggard, 2017; Perri et al., 2016, 2017). It is probably not a case that the volitional control was reduced in the study where the dorsal-medial PFC was inhibited (Perri et al., 2022), while the self-awareness and the self-rated hypnotic depth are among the dimensions affected by the present stimulation which targeted lateral portions of the PFC. As a further confirmation, neurostimulation studies on agency outside of hypnosis revealed that the tDCS over the pre-SMA affected the implicit measures of agency (for a review see Haggard, 2017) while stimulation of the DLPFC altered the feeling of control over voluntary actions (Perri & Perrotta, 2021; Perrotta et al., 2021), but only when participants selected between multiple actions (for a meta-analysis see Khalighinejad et al., 2016). Moreover, it should be noted that despite agency disruption has been described as a key-aspect of the hypnotic experience (Rainville & Price, 2003), the spontaneous emergence of loss of control following a neutral hypnotic induction has received little or no evidence (Cardeña et al., 2013; Polito et al., 2014). At the opposite, the use of hypnotic suggestions targeting loss of control over one's own actions was associated with changes in the agency-related brain activities (Walsh et al., 2015). In other words, sense of agency should be intended as a dynamic multidimensional construct that varies across items and over time (Polito et al., 2014), not “switched off” by hypnotic induction but correlated with hypnotizability and responsiveness to suggestions as shown by previous (e.g., Polito et al. 2013) and the present study. In particular, we observed a positive correlation between the self-rated response to suggestions and the SOARS (mainly the effortlessness factor), as well as between the involuntariness of SOARS and the HSS, but only before tDCS; on the contrary, the HSS was not associated with the self-rated response to suggestions. In fact, the tDCS-induced increase of hypnotizability was not paralleled by an increase in either responses to suggestions or agency measures. These data confirm that there is not a causal relationship between the global index of hypnotizability (as estimated by the HSS; Pekala et al. 2017) and the changes of agency: the latter is only one of the phenomena contributing to the hypnotic experience, but is probably one of the main factors modulating the behavioural response of suggestibility. The neurostimulation of other areas of the agency brain network such as the angular gyrus (see, e.g., Khalighinejad & Haggard 2015) could clarify whether it leads to changes in the feeling of control and pass rates for different items on the traditional behavioural scales of hypnotic suggestibility. Stimulation of posterior areas could also help prevent possible confounding interpretations of changes in the PFC activity: in fact, despite the inhibition of the PFC was identified as a sign of the reduced DN activity during neutral hypnosis (for a meta-analysis see Landry et al., 2017), a greater engagement of PFC was observed when hypnotic suggestions required increased executive control (Huber et al., 2013; Perri et al., 2020a; Zahedi et al., 2017, 2019), thus suggesting the cognitive flexibility (and not the PFC suppression) as a core aspect of hypnotic abilities.

Findings from the present and the previous studies of neurostimulation corroborate the view of hypnotizability as a multidimensional construct (Woody et al., 2005) also associated with state changes in the ability to experience hypnosis. As for the neurocognitive implications, these findings confirm the key-role of the lDLPFC in the hypnotic phenomena, but assumptions of the COLD control theory cannot be fully confirmed. In fact, this model claims that hypnosis would produce a temporary reduction of awareness of one's intentions in thoughts and action (Dienes & Perner, 2007), and that this metacognitive process would be associated with the suppression of the DLPFC also leading to greater suggestibility (Dienes & Hutton, 2013). While the role of the DLPFC in the conscious awareness is undoubted (e.g., Bodovitz 2008), the specific contribution of this region in the agency-mediated alteration of suggestibility remains controversial. In fact, our inhibitory tDCS did not produce any modulation neither in the behavioural response to suggestions nor in the associated feeling of agency, while the Self-awareness dimension of consciousness was reduced. The latter was defined as the measure of “*the extent to which the participant is aware of being aware of their self or whether the participant looses consciousness of himself or is not aware of being aware of himself*” (Pekala and Kumar, 1984). In other terms, changes in volitional control and self-awareness may affect the subjective but not the behavioural experience for which the contribution of suggestion-related brain areas should probably be tested. For example, it could be interesting to observe if the stimulation of motor and sensory areas can enhance the responsiveness to the ideomotor and perceptual items. Moreover, because it becomes increasingly evident that the subjective and the behavioural experience reflect different components of hypnotizability (Acunzo & Terhune, 2021; Facco, 2021; Hung & Barnier, 2005; Perri et al., 2020a), it is necessary for future studies to adopt also phenomenological measures when investigating the variation of consciousness during hypnosis.

In conclusion, this study suggests the NIBS as a promising tool to modify the hypnotic phenomena. In fact, the causal approach of NIBS offers the unique opportunity to test the contribution of different cortical areas on the assessed phenomena, with the double aim to know more about the neurophysiology of consciousness and to produce modification in the interior and exterior experience. Among the limitations of this study there is the scarcity of behavioural measures of suggestibility for which the traditional assessments of hypnotizability such as the Harvard Scale (Shor & Orne, 1962) are more suited. Further, a larger sample would have made it possible to compare the tDCS effects on different classes of hypnotizability. Future studies adopting different montages and methods of stimulation (e.g., online vs offline), and recruiting subjects with high or low hypnotizability could also clarify if the increase of hypnotic responsiveness is enough to produce better responses to clinical procedures adopting hypnosis. In fact, implications of these findings might be manifold: for example, modification of hypnotic experience could be translated into better outcomes for hypnotic interventions like pain management, cognitive and emotional regulation. If so, all forms of hypnotherapy could theoretically benefit from neuroenhancement with consequent benefits for patients, and it may also be possible to broaden the audience of potential hypnotic clients by recruiting subjects who would otherwise be considered refractory to hypnosis. Moreover, even outside of hypnosis this line of research offers important implications for the cognitive and behavioural sciences, as well as for clinical applications of NIBS. In fact, NIBS are indicated as an evidence-based therapy for depression and addictions (for a review see Perrotta & Perri 2022) for which the left DLPFC is usually stimulated (for meta-analyses see Li et al. 2022; Tseng et al. 2021). According to present findings, the efficacy of this approach could lie in increasing Self-awareness, whose reduction is a core aspect of addiction (e.g., Hull 1981) and mood disorders (e.g., Pyszczynski & Greenberg 1987) also associated with motivational states (Boekaerts, 1999) and stress reactivity (Rith-Najarian et al., 2014).

## Funding

This research did not receive any specific grant from funding agencies in the public, commercial, or not-for-profit sectors.

## Declaration of Competing Interest

The authors declare that they have no known competing financial interests or personal relationships that could have appeared to influence the work reported in this paper.
